# Clindamycin-rifampin combination therapy for staphylococcal periprosthetic joint infections: a retrospective observational study

**DOI:** 10.1186/s12879-017-2429-2

**Published:** 2017-05-02

**Authors:** Borg Leijtens, Joris B. W. Elbers, Patrick D. Sturm, Bart Jan Kullberg, Berend W. Schreurs

**Affiliations:** 1 Department of Orthopaedic Surgery, Radboud University Medical Centre, PO Box 9101, 6500 HB Nijmegen, The Netherlands; 2 Department of Medical Microbiology, Radboud University Medical Centre, Nijmegen, The Netherlands; 3 Department of Internal Medicine and Radboud Centre for Infectious Diseases, Radboud University Medical Centre, Nijmegen, The Netherlands

**Keywords:** Clindamycin, Rifampin, Periprosthetic joint infection, Staphylococcus

## Abstract

**Background:**

Staphylococcal species account for more than 50% of periprosthetic joint infections (PJI) and antimicrobial therapy with rifampin-based combination regimens has been shown effective. The present study evaluates the safety and efficacy of clindamycin in combination with rifampin for the management of staphylococcal PJI.

**Methods:**

In this retrospective cohort study, patients were included who received clindamycin-rifampin combination therapy to treat a periprosthetic hip or knee infection by *Staphylococcus aureus* or coagulase-negative staphylococci. Patients were treated according to a standardized treatment algorithm and followed for a median of 54 months.

Of the 36 patients with periprosthetic staphylococcal infections, 31 had an infection of the hip, and five had an infection of the knee. Eighteen patients underwent debridement and retention of the implant (DAIR) for an early infection, the other 18 patients underwent revision of loose components in presumed aseptic loosening with unexpected positive cultures.

**Results:**

In this study, we report a success rate of 86%, with five recurrent/persistent PJI in 36 treated patients. Cure rate was 78% (14/18) in the DAIR patients and 94% (17/18) in the revision group. Five patients (14%) discontinued clindamycin-rifampin due to side effects. Of the 31 patients completing the clindamycin-rifampin regimen 29 patients (94%) were cured.

**Conclusion:**

Combined therapy with clindamycin and rifampin is a safe, well tolerated and effective regimen for the treatment of staphylococcal periprosthetic infection.

## Background

Periprosthetic joint infections (PJI) cause significant morbidity and a considerable claim on the health care resource utilization. Implant-associated infections are typically caused by microorganisms that adhere to the device surface and produce microbial biofilms. Staphylococci account for more than 50% of the periprosthetic joint infections [1]. The treatment is challenging, as the organisms in the biofilm are protected from antimicrobial agents and host responses, and have greater resistance to antimicrobial killing [2, 3]. Rifampin-based combination therapy regimens have been shown effective to eradicate staphylococcal biofims and cure PJI [4]. In the widely-used algorithm proposed by Zimmerli et al. [3] and the IDSA (Infectious Disease Society of America) guidelines [5], rifampin is combined with quinolones, and cure rates of 70-100% have been reported. Although other agents, e.g., betalactams, glycopeptides, minocycline, cotrimoxazole, linezolid, daptomycin, or fusidic acid have been investigated in combination with rifampin, their efficacy is in general inferior or data are anecdotal [4–7]. Clindamycin has been well established as antistaphylococcal therapy, but very few clinical data are available about its use in combination with rifampin for PJI. Therefore, clindamycin has not yet been recommended as an alternative to combine with rifampin to treat PJI in the IDSA guidelines [5]. Clindamycin has been shown to be effective in treatment of osteomyelitis [8], has excellent bioavailability, high levels of penetration into synovial fluid and bone [9, 10], inhibits biofilm formation and bacterial adherence and is well tolerated [11, 12]. In vitro, clindamycin prevents the emergence of rifampin resistance, and the combination displayed synergetic or additive bactericidal activity, as well as favorable cure rates in animal models [13–18]. To date, only 2 case series have reported on the efficacy of an oral clindamycin-rifampin combination therapy regimen for staphylococcal PJI in adults, with a success rate of 70% in 7 cases and 100% in 6 cases [6, 19].

In the present study, we evaluated the efficacy and safety of a clindamycin-rifampin combination therapy regimen for the management of PJI caused by *Staphylococcus* species.

## Methods

### Patients

For this retrospective cohort study, patients who received complete treatment for PJI of the hip (118) or knee (36) in our hospital between January 2004 and June 2010 were eligible. Inclusion criteria were PJI due to *S. aureus* or coagulase-negative staphylococci (CoNS). The isolated staphylococci had to be susceptible in vitro to both clindamycin and rifampin which was determined by automated susceptibility testing using the BD Phoenix™ (BD Diagnostics, Sparks, MD, USA).

### Diagnosis

PJI was diagnosed according to the MSIS (Musculoskeletal infection society) criteria [20]; Presence of 1-2 major criteria and/or three minor criteria. Major criteria are: 1) two positive periprosthetic cultures with phenotypically identical organisms 2) A sinus tract communicating with the joint. Minor Criteria are: 1) Elevated serum C-reactive protein AND erythrocyte sedimentation rate 2) Elevated synovial fluid white blood cell count OR ++change on leukocyte esterase test strip 3) Elevated synovial fluid polymorphonuclear neutrophil percentage 4) Positive histological analysis of periprosthetic tissue 5) A single positive culture. In case of postoperative events, newly isolated microorganisms were typed by standard molecular techniques if necessary.

### Management

Management of PJI was based on the treatment algorithm by Zimmerli et al. [3] and the IDSA Guidelines [5], performed by a multidisciplinary team consisting of an orthopedic surgeon, a microbiologist and an infectious diseases specialist.

### Debridement

DAIR was applied for (a) early postoperative or acute haematogenous infection, with (b) duration of symptoms <3 weeks, (c) a stable implant and (d) if soft tissue was in good condition. The wound was re-opened, deep cultures were taken, debridement was performed and pulse lavage was used with at least 3 l of NaCl 0.9% solution. Gentamicin beads were inserted based on the surgeon’s decision and removed after 2 weeks. No change of mobile parts was performed during surgery.

#### Revision

This group consisted of patients who underwent a revision of (a part of) the prosthesis for presumed aseptic loosening in the absence of positive MSIS criteria preoperatively. In these included cases, two or more perioperative taken cultures showed bacterial growth and retrospectively proved a prosthetic joint infection according to the MSIS criteria. At the revision, debridement was performed and pulse-lavage was used with at least three litres of NaCl 0.9% solution. In case of purulence at the implant site, complete revision was performed. Otherwise, only loose parts of the implant were revised and stable parts were let in situ. So retrospectively, these patients underwent a one-stage revision of all loose parts of the implant for septic loosening. If indicated, bone defects were reconstructed with impaction bone grafting [21]. A cemented prosthesis was inserted using Simplex® bone cement with 500 mg erythromycin and 3.000.000EH colistin (Stryker, Newbury, UK) [22, 23].

### Antimicrobial treatment

Surgical prophylaxis (cefazolin 2 g intravenously) was administered after deep tissue cultures were taken. Post-surgery, initial intravenous therapy was continued for 2 weeks with a betalactam antibiotic, clindamycin or teicoplanin, based on previous cultures if present. Antibiotics were switched to oral withtin 2 weeks. Oral antibiotic regimen consisted of: clindamycin (600 miligrams three times daily) and rifampin (450 miligrams twice daily) for a minimum of 3 months, once the isolate had been identified and found susceptible to both drugs.

### Follow-up

Patients were clinically and radiographically evaluated at the outpatient clinic at 6 weeks, 3, 6 months, and then yearly. Follow-up was continued until April 2015 with a median of 54 months (range 1-120).

### Definitions

*Cure*: (a) no clinical, radiological or laboratory signs of infection at the latest follow-up, with a minimum of 2 years after re-implantation, (b) proven negative perioperative cultures in case of reoperation for other reasons than infection or (c) positive cultures yielding a different microorganism after an uneventful follow-up of at least 2 years. *Failure* (persistence/recurrence of infection): (a) persistent or recurrent signs of infection in the first 2 years after debridement or one-stage revision, regardless the microorganism in newly obtained cultures or (b) isolation of the same microorganism as found at the initial treatment at any reoperation on the affected side during follow-up.

#### Statistical analysis

Graphpad Prism^©^ version 11.0 was used for creation of the Kaplan-Meier curves. Data were analysed according to the intention-to-treat principle. No *p*-values were calculated since goal of this study was to evaluate the outcome of this treatment protocol and not to compare the outcome of DAIR versus one-stage revision.

## Results

### Study population

Between January 2004 and June 2010, 154 patients received treatment for PJI of the hip (118 patients) or knee (36 patients). PJI of the hip was treated with DAIR (38 patients), one-stage revision (31), two-stage revision (33) or prosthesis removal without prosthesis replacement (16). Patients with total knee arthroplasty infection were treated with DAIR (18 patients), one-stage revision (2), two-stage revision (13), arthrodesis (1) or amputation (2). Of the 89 patients treated with DAIR or one-stage revision, 51 patients had a staphylococcal PJI. Fifteen of those patients were excluded due to the following reasons: potential rifampin drug interaction (1); possibility of iMLS_b_ phenotype resistance (1) [24]; clindamycin (5) or rifampin (5) resistance (in 9 out of these 10 patients, the microorganism was resistant to quinolones as well). Three patients were excluded based on protocol violation.

A total of 36 patients (23%) were treated with the clindamycin-rifampin combination therapy after DAIR (18 patients) or revision (18 patients). Demographic characteristics of all patients are shown in Table 1. Duration of symptoms before surgery was <3 weeks in all cases in the debridement group. There were six patients with an acute haematogenous PJI in the DAIR group. Duration of symptoms was >3 weeks in all patients in the revision group (4-79 weeks). Characteristics of all PJI are presented in Table 2.

**Table 1:** Patients with periprosthetic *Staphylococcus* infection receiving clindamycin-rifampin combination therapy

	Debridement and retention	Revision
	n (%)	n (%)
Number of patients	18	18
Age, years; median (range)	71 (39 – 89)	58 (30 – 87)
BMI^a^, kg/m^2^; median (range)	26 (20 – 32)	25 (19 – 35)
Gender, male	12	13
Prosthesis site		
Hip	13	18
Knee	5	0
Indication for prosthesis		
Primary arthrosis	5	3
Secondary arthrosis		
Childhood hip disease	1	4
Post traumatic	3	2
Osteonecrosis	1	1
Rheumatoid arthritis	1	0
Hemophilia	1	0
Revision arthroplasty	5	6
Unknown	0	2
Femoral neck fracture	1	0
Risk factors for PJI^b^ / comorbidity		
Previous hip/knee surgery before primary THA/TKA	10	10
Immune suppression	1	2
Previous PJI	1	2
Diabetes Mellitus	2	2
Obesity (BMI^a^ > 30 kg/m^2^)	1	3
ASA^c^ score; median (range)	2 (1 – 3)	2 (1 – 3)

^a^BMI, Body Mass Index

^b^PJI, Periprosthetic Joint Infection

^c^ASA, American Society of Anesthesiologists; THA, total hip arthroplasty; TKA, total knee arthroplasty

**Table 2:** Characteristics of periprosthetic infection

	Debridement and retention (n)	Revision (n)
Number of patients	18	18
Manifestation of infection		
Early (≤ 3 months)	12	3
Delayed (3-24 months)	2	2
Late (≥ 24 months)	4	13
Age of implant, weeks; median (range)	7 (1 – 442)	263 (29 - 862)
Referred from another hospital	0	9
Perioperative cultures		
Methicillin-susceptible *Staphylococcus aureus*	11	2
Methicillin-resistant *Staphylococcus aureus*	1	0
Coagulase-negative staphylococci	5	14
Polymicrobial	1	2
Number of positive cultures per patient; median (range)	3 (1 – 7)	6 (2 – 9)

### Surgical treatment

In the debridement group, gentamicin beads were inserted in ten cases (56%) and removed at a second debridement. Median duration of surgery was 35 min (range, 18-94). In the revision group, complete revisions was performed in six cases and partial revisions in 12 cases (eight acetabulair and four femoral), with a median duration of surgery of 171 min (90-290). In 15 patients (83%), bone defects were reconstructed with impaction bone grafting (Table 3).

**Table 3:** Characteristics of surgical and antimicrobial therapy

	Debridement and retention *n* = 18 n	Revision *n* = 18 n	Total *n* = 36 n
Surgery			
Duration of surgery, minutes; median (range)	34 (18-94)	1 71 (90-290)	91 (18-290)
Gentamicin beads used	9 (53)	-	
Bone impaction grafting	-	15 (83)	
Complete / partial revision	-	6 (33) / 12 (67)	
Antimicrobial therapy			
Prior intravenous antibiotics	12	9	21
Duration of iv therapy, days; median (range)	11 (2 - 56)	12 (2 - 15)	11 (2-56)
Duration of iv + oral antibiotic therapy, days; med (range)	101 (31 – 239)	92 (80 – 139)	98 (31-239)
< 90 days	3	2	5
Hip PJI^a^, median (range)	98 (31-146)	92 (80-139)	95 (31-146)
Knee PJI, median (range)	182 (117-239)	-	
Rifampin dose reduction (300 mg twice daily)	3	3	6
Clindamycin-rifampin discontinuation	3	2	5
Due to:			
Comorbidities	2	1	3
Side effects			
Allergy/Rash	2	1	3
Nausea	2	1	3
Diarrhea	0	2	2
Treatment outcomes			
Failures	4	1	5
Successfully treated	14 (78%)	17 (94%)	31 (86%)
Patients completed clindamycin-rifampin regimen	15	16	31
➜ Successfully treated	14 (93%)	15 (94%)	29 (94%)

^a^PJI, Periprosthetic Joint Infection

### Antimicrobial treatment

Surgical prophylaxis (cefazolin 2 g intravenously) was administered in all patients. Table 3 summarizes the antibiotic regimen in our patient group. The minimum duration of oral clindamycin-rifampin treatment was 70 days in the debridement group and 66 days in the revision group, both after initial intravenous therapy.

### Side effects and treatment discontinuation

Five of the 36 patients (14%) discontinued clindamycin due to side effects after 3-41 days (Table 3). In the debridement group, one patient switched to levofloxacin because of fatigue and loss of appetite, one patient to flucloxacillin because of rash and one patient to ciprofloxacin due to an allergic reaction to clindamycin. In the revision group, one patient switched to teicoplanin and one patient to ciprofloxacin, both because of diarrhea, without demonstration of *Clostridium difficile* toxin. The latter patient experienced the same severity of diarrhea on ciprofloxacin as on clindamycin, but was able to complete antibiotic treatment. All five patients continued with rifampin treatment.

Dose reduction of rifampin (to 300 mg bid) was applied in six patients (17%), due to side effects (three patients) and co morbidities (three patients) (Table 3). All of these patients were treated successfully.

### Treament outcome

**Fig. 1:**
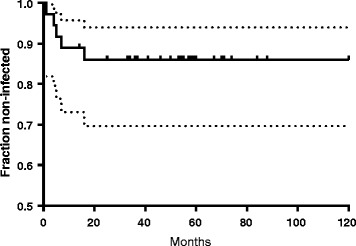
Probability of cure. Kaplan-Meier survival curves of the total group of 36 patients. Tick marks indicate patients censored due to loss of follow-up or infection-unrelated events. Dotted lines indicate confidence intervals

As shown in Table 3, 86% of the patients (31 out of 36 patients) were treated successfully. The Kaplan-Meier survival curve of time to treatment failure is shown in Fig. 1. In patients completing the clindamycin-rifampin regimen, cure rate was even higher; 29 out of 31 patients (94%).

Characteristics of treatment failures are shown in Table 4. In three of the five failures clindamycin was switched to an alternative antibiotic due to side effects; all after DAIR. The two other failures had a recurrent PJI despite a complete clindamycin-rifampin treatment; one in the DAIR group, the other in the revision group.

**Table 4:** Failures

Case	Surgical treatment	Time from primary prosthesis to PJI (months)	Cultures	Antibiotic treatment	Time to recurrence of infection after ceasing AB (days)	Regimen	Cultures at reoperation
1	DAIR^a^	49	*S. aureus*	Clinda^a^/ rifampin 89 days	16	Suppressive clindamycin	-
2	DAIR	101	CoNS + *S. mitis*	Clinda / rifampin 41 days	28	Suppressive doxyclin	-
				Levoflox^b^ / rifampin 86 days			
3	DAIR	1.5	S. *aureus*	Clinda / rifampin 19 days	0 (infection persisted)	Girdestone	Negative
				Fluclox^c^ / rifampin 6 days			
4	DAIR	11	S. *aureus*	Clinda / rifampin 12 days	320	two stage revision	S. *aureus*
				Ciproxin^d^ / rifampin 83 days			
5	Revision	48	S. *capitis*	Clinda / rifampin 92 days	0	Girdlestone	S. *capitis*

*PJI* prosthetic joint infection; *AB* antibiotics; *DAIR* debridement and implant retention; *CoNS* coagulase negative Staphylococci

^a^Clindamycin

^b^Levofloxacin

^c^Flucloxacillin

^d^Ciprofloxacin

Three patients died due to non-orthopedic causes without any signs of infection after 36-84 months of follow-up. Four patients underwent a re-operation of the affected side. In two of these patients cultures at reoperation were negative. One patient experienced a periprosthetic fracture 4.5 years after one-stage revision for *S. aureus* infection. At re-operation two different strains of CoNS were isolated, both susceptible to clindamycin and rifampin. In another case, late prosthetic infection occurred at 54 months after complete clinical cure of PJI with *S. epidermidis*. A Girdlestone procedure was performed with collection of 12 cultures from 4 different areas. Again *S. epidermidis* was cultured that was now clindamycin and rifampin resistant. The stored isolates of the initial infection and the infection after 54 months were unrelated as established by fingerprinting using Raman spectroscopy, SpectraCell RA^®^ method (River Diagnostics, Madison, WI) [25].

## Discussion

In the present report, we describe 36 patients with culture-confirmed staphylococcal PJI treated with the combination of clindamycin and rifampin, leading to a probability of cured infection of 86% after >4 years of follow-up and 94% in patients able to complete clindamycin-rifampin therapy. These results are comparable to those reported in previous research combining rifampin with different antibiotics. Zimmerli et al. describe a 100% success rate after debridement and ciprofloxacin-rifampin combination therapy with a median follow-up of 35 months in 18 patients of whom 12 completed the treatment regimen [26]. In four patients (22%) rifampin dose was reduced and two patients (11%) discontinued rifampin or ciprofloxacin. Among these six drop outs there were two failures. Another retrospective cohort study presents a 77% infection free survival after 24 months in 43 methicillin resistant staphylococcal PJI treated with rifampin and fucidic acid after DAIR [27]. A large multicenter cohort study presents a 55% success rate in 328 PJI treated with rifampin plus any other antibiotic after DAIR [28].

The side effects of clindamycin-rifampin combination therapy were limited in our study. Treatment was discontinued in 5/36 (14%) patients, due to side effects. This adverse event rate is similar to that reported for other regimens, such as 14% (diarrhea) during rifampin-levofloxacin and 16% (nephrotoxity and diarrhea) during levofloxacin alone [29]. In the study where rifampin and fucidic acid were combined, 3 patients (7%) experienced side effects.

Our treatment protocol slightly deviated from the original algorithm published by Zimmerli et al. [3]. First, not all patients were treated with 2 weeks of intravenous antibiotics initially. However, due to its excellent bioavailability, clindamycin seems appropriate for early oral therapy resulting in reduction of hospital stay and costs. Second, some patients with PJIs of the knee were treated for a limited period of time (17-34 weeks), whereas guidelines recommend antibiotic treatment for 6 months in case of periprosthetic knee infection [3]. The evidence supporting a long duration of treatment is limited [30], and 3 months of treatment may be sufficient similar to periprosthetic hip infection [31, 32]. Third, the optimal dose of rifampin is unknown, and results from other studies suggest that rifampin 600 mg/24 h is as effective as 900 mg/24 h [6, 33]. Therefore, in 6 patients, rifampin dose reduction to 600 mg/24 h was administered due to side effects, drug interaction or co-morbidity. All were treated successfully. This dose reduction regimen is supported by recently published data on rifampin dosage and frequency in PJI [33].

This study has several limitations. First, patients were not randomized to receive clindamycin-rifampin or a comparator regimen. However, the high success rate suggests that the present regimen may be as effective as quinolone-rifampin combination therapy, warranting a prospective, randomized controlled trial. A second limitation is the limited number of patients in the present study cohort. Also, we regret the heterogeneity of the cohort; In the DAIR group, gentamicin beads were inserted based on the surgeon’s decision and removed after 2 weeks. In the revision group, complete revisions were performed in six cases and partial revisions in 12 cases. Furthermore, 15 of the 18 cases had a significant bone defect which was reconstructed with impaction bone grafting.

While performing this study, two articles on the pharmacokinetics of the clindamycin-rifampin combination regimen were published [34, 35]. Bernard et al. and Cruris et al. reported a dramatic reduction of clindamycin serum concentration in patients receiving clindamycin with rifampin compared to patients receiving clindamycin without rifampin. This reduction could be explained by the induction of cytochrome P450 by rifampin. Clindamycin is metabolized through CYP3A4, a member of the cytochrome P450 system. Despite their low numbers of patients these studies found a significant decline of serum concentration levels of clindamycin, below therapeutic range. However, Bernard et al. report a 82% success rate in 11 patients receiving clindamycin with rifampin for a staphylococcal osteo-articular infection. Cruris et al. report a 100% success rate in 7 patients receiving clindamycin with rifampin. Zeller et al. analysed 24 patients receiving continuous iv clindamycin therapy combined with rifampin. Serum clindamycin concentrations declined after rifampin administration but not below therapeutic concentration [12]. Specific success rate of this group is not documented.

We emphasise that pharmacokinetics and optimal serum concentration levels of this combination regimen needs to be investigated. Another recently published article describes the termination of a prospective study due to a dramatic decline in fusidic acid plasma concentration when used in combination with rifampin [36]. The likely explanation for this decline would again be the induction of CYP3A4 by rifampin.

The strengths of the present study are the inclusion of culture-proven PJIs of the hip and knee only, excluding other implanted devices. In addition, we report high success rates in partial one-stage revisions in preoperatively considered aseptic loosening which turned out to be septic. This indicates that this antibiotic combination therapy is capable of eradicating a PJI without removal of all devices. Furthermore, during the entire study period, a standardized multidisciplinary approach according to international treatment algorithms was used.

## Conclusion

Clindamycin-rifampin combination therapy results in a high success rate in the treatment staphylococcal PJI. The present findings warrant a randomized controlled trial to assess whether this combination regimen is a welcome addition to our arsenal against staphylococcal PJI.

## Abbreviations

CoNS: Coagulase-negative staphylococci
DAIR: Debridement and implant retention
IDSA: Infectious Disease Society of America
MSIS: Musculoskeletal infection society
PJI: Periprosthetic joint infections
